# Genome sequence of *Streptomyces* BM cluster phage Frankenweenie

**DOI:** 10.1128/MRA.00592-23

**Published:** 2023-10-13

**Authors:** Katherine E. Cleary, Charles Pelagalli, Marly Cassford, Nathan Berry, Elizabeth Aguas, Brandon Kim, Tagide deCarvalho, Deborah Jacobs-Sera, Steven M. Caruso, Kathleen Cornely

**Affiliations:** 1 Department of Chemistry and Biochemistry, Providence College, Providence, Rhode Island, USA; 2 Department of Biological Sciences, University of Maryland, Baltimore County, Baltimore, Maryland, USA; 3 Department of Biological Sciences, University of Pittsburgh, Pittsburgh, Pennsylvania, USA; Loyola University Chicago, Chicago, Illinois, USA

**Keywords:** phage, *Streptomyces*, genome analysis

## Abstract

Frankenweenie is a newly isolated bacteriophage that infects *Streptomyces scabiei* RL-34. Frankenweenie was discovered in Gaithersburg, MD, and has 366 genes comprising a 200,048-bp genome. Frankenweenie is grouped in cluster BM and is predicted to possess a unique tailspike protein that potentially widens its host range.

## ANNOUNCEMENT

*Streptomyces* phage Frankenweenie was isolated using *Streptomyces scabiei*, the causative agent of the potato common scab. Bacteriophages targeting this pathogen have shown promise in controlling the scab, which causes potato lesions, resulting in economic losses to populations depending on the tuber as a food source. Phage-mediated biocontrol provides an environmentally sensitive strategy for controlling the pathogen, in contrast to traditional methods such as soil treatment and foliar sprays (1) which are insufficient for controlling the disease.

**Fig 1:**
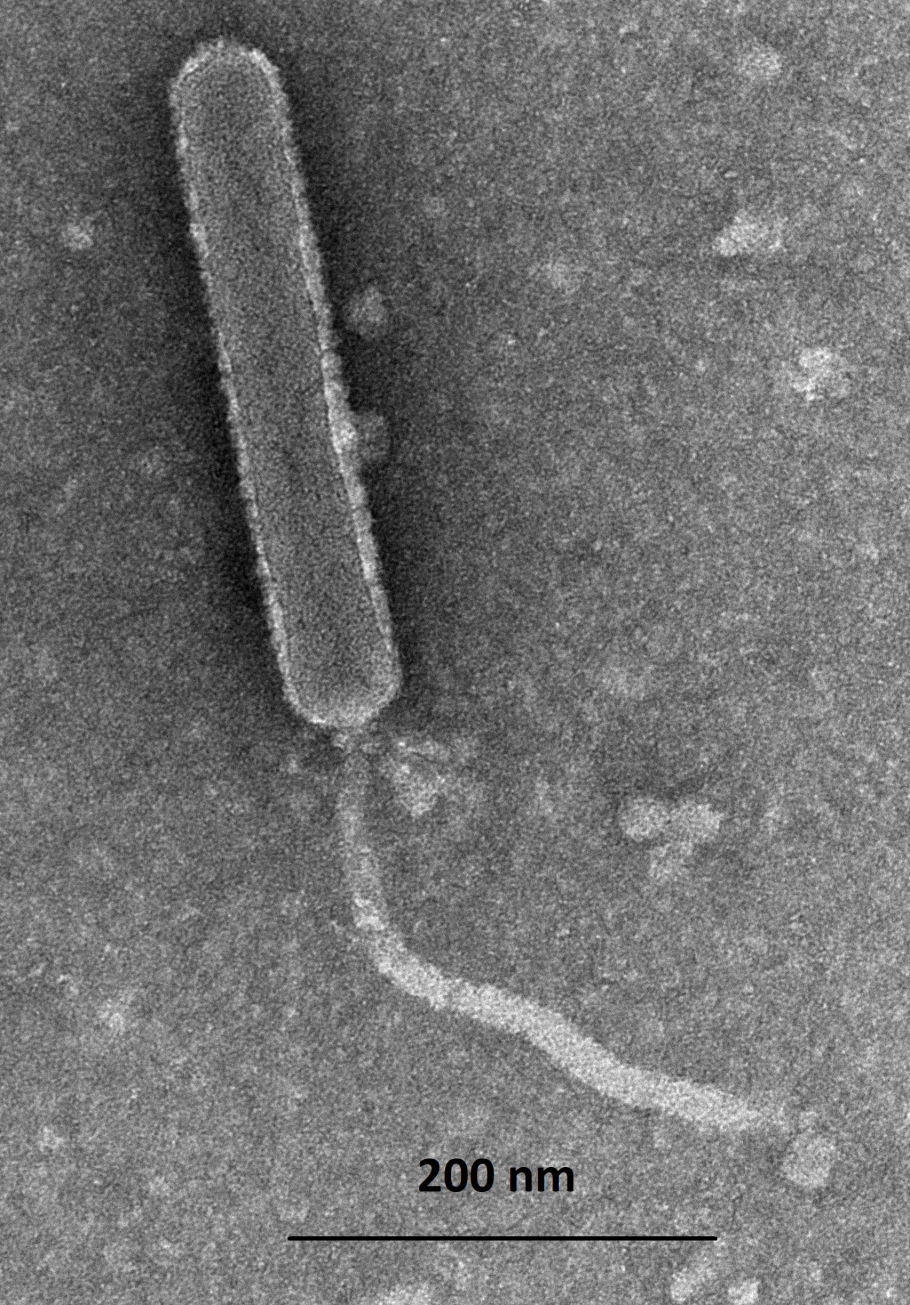
Image of Frankenweenie taken with a Hitachi ×120k transmission electron microscope. Electron microscopy revealed that Frankenweenie exhibits unusual siphovirus morphology with prolate heads. The capsid is 327–334 nm in length and 51–59 nm in width. The large capsid is consistent with the exceptionally large genome for this phage. Frankenweenie has a flexible tail 291–311 nm long.

Frankenweenie was isolated and characterized through the participation of our institutions in the Science Education Alliance-Phage Hunters Advancing Genomics and Evolutionary Sciences (2). The phage was discovered and purified at the University of Maryland, Baltimore County and annotated at Providence College. Frankenweenie was isolated from soil collected in Gaithersburg, MD, using standard methods (3). Soil samples were flooded with 10 mM Tris, 10 mM MgSO_4_, 1 mM CaCl_2_, 68.5 mM NaCl pH 7.5 buffer and centrifuged; the supernatant was filtered (0.22 µm pore) and plated on nutrient agar (BD Difco) supplemented with 10 mM MgCl_2_, 8 mM Ca(NO_3_)_2_, and 0.5% glucose, overlaid with tryptic soy top agar (BD) inoculated with *Streptomyces scabiei* RL-34, and then incubated for 24 hours at 30°C to yield plaques, which then underwent three rounds of purification. Negative-stain transmission electron microscopy revealed that Frankenweenie exhibits a siphovirus morphology with prolate heads (Fig. 1). Phage DNA was isolated from high-titer lysates using a Wizard genomic DNA purification kit (Promega) and sequenced at the University of Pittsburgh (Table 1). The untrimmed reads were assembled into a single contig using Newbler v2.9 (4) and verified using Consed v29.0 (5).

**TABLE 1:** Sequencing, phage, and genome characteristics

Parameter	Phage data
Sequencing	
Sequencing instrument	Illumina MiSeq (v3 reagent)
Library prep kit	NEB Ultra II
Number of reads	2,046,921
Length of reads (bp)	150 single end reads
Shotgun coverage (×)	1,482
Phage characteristics	
Genome length (bp)	200,048
Character of genome ends	Direct terminal repeat
Direct terminal repeat length	1,038 bp
GC content	66.5%
Sample characteristics	
Collection date	9 September 2021
Collection location coordinates	39.10871 N, 77.219247 W
Isolation temperature	30°C
Capsid length (nm)	327–334
Number of particles measured	*n* = 3
Mean ± std (nm)	331 ± 3
Capsid width (nm)	50–59
Number of particles measured	*n* = 3
Mean ± std (nm)	56 nm ± 4
Tail length (nm)	291–312
Number of particles measured	*n* = 3
Mean (nm)	303 nm ± 11
Genome characteristics	
Total number of genes	366
Number of genes with identified functions	64
Number of genes without identified functions	287
Number of orphams	32
Number of tRNAs	15

DNA Master v5.23.6 (6) was used to perform the genome annotation. GeneMark v2.5 (7), Glimmer v3.02 (8), and Starterator v1.1 (9) were used to determine start sites. Protein-coding gene functions were determined using HHpred (PDB, UniProt, Pfam databases) (10), BLASTp v2.9 (11), and genome organization using Phamerator (12). Membrane proteins were identified using TMHMM v2.0 (13) and TOPCONS (14). tRNAs were identified using tRNAscan-SE (15) and Aragorn (16). Default parameters were used unless otherwise indicated. Frankenweenie’s chromosome is 200,048 bp with a 66.5% GC content and 351 predicted protein-coding genes, of which 64 have identifiable functions. Frankenweenie was assigned to cluster BM with other phages in the cluster (JustBecause [MH744418] and Satis [M576962]) sharing >50% nucleotide identity and/or >35% gene content similarity (17 – 23). Frankenweenie possesses no integrase or repressor and is unlikely to adopt a temperate lifestyle.

Frankenweenie differs considerably from phages in the BM cluster in length and number of orpham genes. Of note is a 3,026-bp orpham likely to be a tailspike protein. This protein is similar to proteins expressed in bacteriophages CBA120 (24), which infects *Escherichia coli*, and SF6 (25), which infects Gram-negative bacteria. Tailspike proteins are involved in host recognition and may bind and modify cell surface structural proteins (26). The additional tailspike protein in Frankenweenie’s genome might allow it to infect a wider range of hosts.

## Data Availability

The complete genome sequence accession number and raw sequencing reads of *Streptomyces* phage Frankenweenie are available with the accession number OQ921725 and the SRA number SRX16768877.
